# The cellular story of dishevelleds

**DOI:** 10.3325/cmj.2014.55.459

**Published:** 2014-10

**Authors:** Anja Kafka, Sandra Bašić-Kinda, Nives Pećina-Šlaus

**Affiliations:** 1 Laboratory of Neuro-oncology, Croatian Institute for Brain Research, University of Zagreb School of Medicine, Zagreb, Croatia; 2 Department of Biology, University of Zagreb School of Medicine, Zagreb, Croatia; 3 Division of Hematology University Hospital Center Zagreb and University of Zagreb School of Medicine, Croatia

## Abstract

Dishevelled (DVL) proteins, three of which have been identified in humans, are highly conserved components of canonical and noncanonical Wnt signaling pathways. These multifunctional proteins, originally discovered in the fruit fly, through their different domains mediate complex signal transduction: DIX (dishevelled, axin) and PDZ (postsynaptic density 95, discs large, zonula occludens-1) domains serve for canonical beta-catenin signaling, while PDZ and DEP (dishevelled, Egl-10, pleckstrin) domains serve for non-canonical signaling. In canonical or beta-catenin signaling, DVL forms large molecular supercomplexes at the plasma membrane consisting of Wnt-Fz-LRP5/6-DVL-AXIN. This promotes the disassembly of the beta-catenin destruction machinery, beta-catenin accumulation, and consequent activation of Wnt signaling. Therefore, DVLs are considered to be key regulators that rescue cytoplasmic beta-catenin from degradation. The potential medical importance of DVLs is in both human degenerative disease and cancer. The overexpression of DVL has been shown to potentiate the activation of Wnt signaling and it is now apparent that up-regulation of DVLs is involved in several types of cancer.

Wnt proteins initiate three distinct signaling pathways − the canonical, non-canonical or planar cell polarity (PCP), and Wnt-Ca^2+^ pathway. The members of the dishevelled (dsh/DVL) protein family are considered to be critical components of Wnt signaling, which are transducing signal into three different cellular routes (1-5). The mechanism how DVL activates distinct downstream pathways has been elucidated recently (6,7), therefore this review attempts to synthesize and explain the current knowledge on DVLs function in Wnt signaling.

Among three Wnt signaling cascades, the canonical Wnt signaling pathway is one of the basic mechanisms of the cell signaling, critical for embryonic development and adult tissue homeostasis, and is widely conserved in the animal kingdom. It is activated by binding of different Wnt ligands (19 were identified in humans) to specific receptors (1). Through several cytoplasmic relay components, the signal is subsequently transduced to beta-catenin. As a consequence, beta-catenin levels raise, and it enters the nucleus to activate transcription of Wnt target genes (2). In the nucleus, beta-catenin finds a partner, a member of the DNA binding transcription factor family LEF/TCF (lymphoid enhancer factor/ T cell factor) (2). Target genes for beta-catenin/LEF/TCF encode c-myc, N-myc, c-jun, and cyclin D1, explaining why constitutive activation of the Wnt pathway can lead to cancer (2,8).

The pathway is inactive when the levels of beta-catenin are kept low. This is achieved by beta-catenin’s degradation in a multiprotein destructive complex consisting of AXIN, APC (adenomatous polyposis coli), CK1 (casein kinase 1), and GSK3β (glycogen synthase kinase 3 beta). This results in beta-catenin phosphorylation, ubiquitination, and finally its degradation in the proteasome (Figure 1). The components of beta-catenin destruction complex represent negative regulators of the pathway. The activation of Wnt signaling pathway also happens in case APC, AXIN, and other components of beta-catenin destruction complex are mutated and non functional (2,8-10).

**Figure 1:**
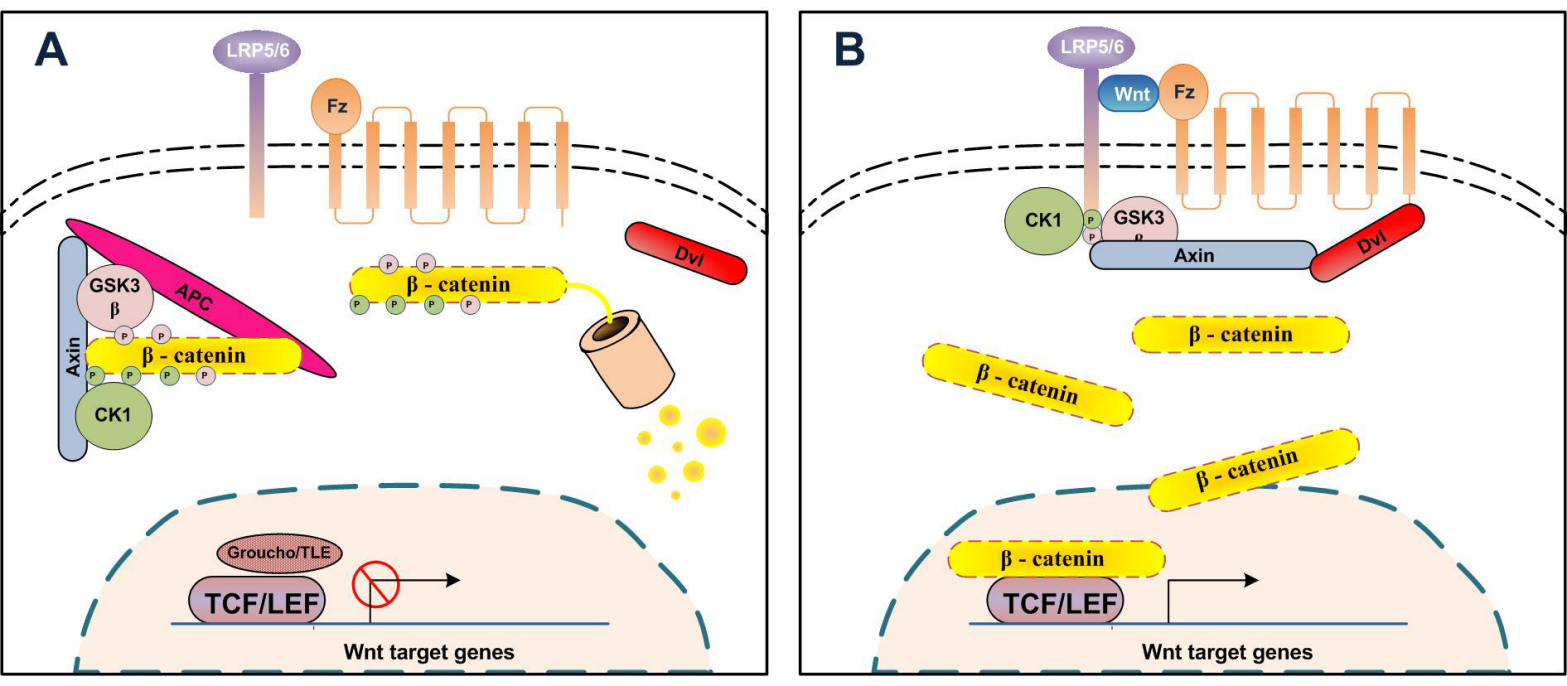
Wnt signaling pathway and its key components. (**A**) In the absence of Wnt, beta-catenin destruction complex consisting of AXIN1, adenomatous polyposis coli (APC), casein kinase 1 (CK1), and glycogen synthase kinase 3 beta (GSK3β) is formed. This results in beta-catenin phosphorylation, ubiquitination, and degradation in the proteasome. (**B**) Binding of Wnt ligands to receptor complex consisting of Frizzled (Fz) and low density lipoprotein receptor-related protein 5 and 6 (LRP5/6) results in recruitment of DVL to the membrane by binding to Fz and AXIN. This disables the formation of beta-catenin destruction complex, allowing beta-catenin to accumulate in the nucleus where it activates Wnt target genes upon binding to lymphoid enhancer factor/ T cell factor (LEF/TCF).

DVL is considered to be a key regulator that rescues cytoplasmic beta-catenin from degradation. Binding of Wnt signaling molecule to membrane receptors activates DVL (2,4). The receptors of the Frizzled (Fz) family are transmembrane seven-pass molecules that work in collaboration with their co-receptors LRP5 and LRP6 (low density lipoprotein receptor-related protein 5 and 6) forming a membrane receptors complex. DVL is recruited to the membrane and it comes in direct contact with the Fz receptor (11). This interaction is essential for phosphorylation of Fz co-receptors LRP5/6 by phosphokinases GSK3β and CK1. Phosphorylation activates LRP5/6 co-receptors, which beside kinases also bind AXIN by the LRP’s cytoplasmic tail. As a consequence, AXIN is recruited to the plasma membrane and it can no longer be a part of beta-catenin destruction complex, so the complex cannot be formed. Besides being able to bind to the co-receptors, AXIN can also bind to DVL. In this way DVL inhibits the activity of AXIN in the destruction complex (8,12). Wnt signal triggers the recruitment of AXIN either to LRP5/6 or to DVL bound to Fz receptors. This leads to the beta-catenin accumulation in the cytoplasm, its consequent nuclear translocation, activation of LEF/TCF transcription factors, and expression of target genes (2-4,6,13-15).

## Why is dishevelled in the center of Wnt signaling?

Dishevelled (dsh) is a multifunctional phosphoprotein originally discovered in the fruit fly *Drosophila melanogaster*. In *Drosophila*, a single *dsh* gene is expressed and it is required for proper development. In contrast, three *dsh* homologue genes (*DVL1*, *DVL2,* and *DVL3*) have been identified in humans showing a high degree of similarity. *DVL1* gene is located at 1p36 locus, and its protein is 695 amino acids long; *DVL2* gene is located at 17p13.1, and its protein is 736 amino acids long; and *DVL3* gene is located at 3q27, and its protein is 716 amino acids long (16-18). The experiments in knockout mice have indicated that each mammalian DVL protein product is able to function cooperatively as well as uniquely (14). DVL2 is the most abundant of the three members (14). Two isoforms of human DVL1 and two of DVL3 are produced by alternative splicing. DVL interacts with more than 50 binding proteins in the cytoplasm and in the nucleus (16).

**Figure 2:**
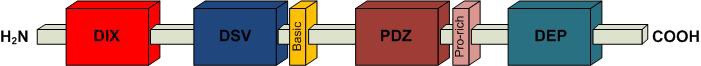
Dishevelled protein structure with conserved domains and regions: DIX (dishevelled, axin) domain, DSV (dishevelled) domain, the basic region, PDZ (postsynaptic density 95, discs large, zonula occludens-1) domain, proline-rich region, and DEP (dishevelled, Egl-10, pleckstrin) domain.

All dsh/DVL proteins (ranging from nematodes to humans) possess three conserved domains: an aminoterminal DIX (dishevelled, axin), a central PDZ (postsynaptic density 95, discs large, zonula occludens-1), and a carboxyl-terminal DEP (dishevelled, Egl-10, pleckstrin) domain (19). In addition DVL also contains another two regions harboring positively charged amino acid residues. The first is called the basic region and is comprised of conserved serine and threonine residues stretching between the DIX and PDZ domains and the second is the proline-rich region, termed SH3 (*src* homology 3) binding domain, which is situated downstream of PDZ (16). The proposed peptide structure is implicated to mediate protein-protein interaction, and thus DVL likely serves as an adapter molecule (4,5). Dillman et al (5) have reported that there is a fourth conserved domain in DVL, called DSV or dishevelled domain, but its functional importance is still unclear (Figure 2).

The N-terminal DIX domain extends in humans for 85 amino acids for DVL1, 83 for DVL2, and 82 for DVL3, and is also present in the AXIN protein, which seems to be a scaffolding factor for Wnt signaling (15,16). DIX domain is necessary but not sufficient for the DVL-AXIN interaction and some other sequences located near to the DIX domain may be requisite. The domains do not interact directly with each other (4). As mentioned previously, DVL can bind AXIN and inhibit its activity (3), thus dissociating the axin-assembled beta-catenin destruction complex by displacing AXIN, or by recruiting another protein called FRAT (frequently rearranged in advanced T-cell lymphomas). Phosphorylated DVL has a high affinity for FRAT, and this binding also induces the disintegration of beta-catenin destruction complex and the activation of the pathway (20). Possibly the interaction of DVL1 with FRAT will cause a conformational change of the degradation complex that phosphorylates beta-catenin.

The central PDZ domain is 73 amino acids long in all three human homologs and it provides a docking site for protein kinases, phosphatases, and adaptor proteins. The proteins that bind to the PDZ domain are best known for their roles in submembranous receptor assembly, where they integrate signaling molecules into larger complexes with subsequent signal transduction (4). The direct interaction of PDZ domain with the Fz through the conserved C-terminal cytoplasmic Fz sequence is essential in transduction of the signal from Fz to the downstream components of the Wnt pathway. It is known that Fz-Dvl and Dvl-Axin protein interactions are relatively weak but dynamic (11).

DEP domain located between PDZ domain and C-terminal region of DVL protein consists of 75 amino acids in all three human homologs. The same polypeptide motif can also be found in signaling factors such as the regulator of G-protein signaling (RGS) protein family, which harbors conserved, catalytic RGS domains as well as DEP domains in their N termini (1). It has been shown that DEP domain enables protein-protein interaction between DVL and DAAM1 (dishevelled associated activator of morphogenesis 1), a formin-homology protein involved in actin polymerization. By mediation of basic residues in DEP domain, DVL binds to membrane lipids during planar epithelial polarization (21).

Considering its hub position in Wnt signaling (4), it is not surprising that domains of DVL proteins contain binding sites for a large number of different proteins, including several kinases. Simply speaking, DVL proteins use different domains through which they mediate complex signal transduction: DIX and PDZ domains are crucial for cannonical beta-catenin signaling, while PDZ and DEP domains are critical for PCP signaling by mediation of the cytoplasmic-to-membrane translocation (1). Recent studies (22) have given more weight to the claim that DIX domain can also be included in non-canonical PCP signaling and DEP domain can affect beta-catenin signaling (22,23). The cellular pool of available DVL is limited, meaning that activation of one pathway makes DVL unavailable in other locations to activate the other pathways. This means that activation of the canonical Wnt pathway could down-regulate non-canonical Wnt signaling, and *vice versa* (24).

## Dishevelled nuclear shuttling

The subcellular localization of DVL is proposed to be causative of the choice of different Wnt pathway routes. Literature data suggest that there are two cellular pools of DVL: one translocates to the nucleus to mediate the canonical signaling while the other remains in the cytoplasm or goes to the plasma membrane and mediates both canonical and non-canonical signaling (1,3,4). Its nuclear localization, which is required for the canonical Wnt beta-catenin signaling, also suggests that the involvement of DVL in Wnt signaling is more complex than previously thought. Regulation of protein shuttling into and out of the nucleus is influenced by the activity of nuclear localization signals (NLS) and nuclear export signals (NES) (25). Wnt signaling utilizes DIX and PDZ domains of DVL to induce the stabilization of cytosolic beta-catenin. When DVL is in the nucleus, it interacts with phosphorylated c-jun and nuclear beta-catenin and mediates the formation of a functional complex consisting of DVL-c-jun-beta-catenin-TCF. Through TCFs sequence-specific DNA binding domain (HMG box), newly formed complex binds on the promoter of Wnt target genes and regulates gene transcriptional activity. Formation of this quaternary functional complex was proposed by Gan et al (26) and it may suggest the transcriptional function of DVL in the nucleus. How DVL’s voyage to the nucleus is regulated remains not completely clear (4,27). In contrast, DVL in the cytoplasm moves to the plasma membrane where it forms large molecular supercomplexes (ie, signalosomes) consisting of Wnt-Fz-LRP5/6-DVL-AXIN necessary for the transmission of signals from the receptor to downstream effectors (22,28). Beside beta-catenin and DVL, many components of the canonical signaling pathway such as APC, AXIN1, and GSK3β appear to traffic between the cytoplasm and the nucleus (27).

## Regulation of DVL activity

To understand the function of DVL's domains and regions several studies have been reported in experimental models (2,3). In spite of a multitude of protein interactions, DVL has no known enzymatic activity. Positive regulation of DVL activity is achieved by phosphorylation of the protein (4). Upon binding of Wnt molecule to receptors, extensive phosphorylation of DVL is induced. Kinases and other factors involved in this hyperphosphorylation event include casein kinase 1 (CK1), casein kinase 2 (CK2), PAR1, and β-arrestin (29,30). The phospho-residues are located along the DVL protein, including the conserved domains.

Negative regulation of DVL activity is accomplished through polyubiquitination by DVL-interacting proteins such as KLHL12, NEDL1, Dapper1, Prickle1, and inversin. Protein phosphatase 2A (PP2A) is also involved in the regulation of DVL activity. It can have a positive or negative influence, depending on which regulatory subunit realizes the binding to DVL (1,4,7,8). Even deubiquitination of DVL by deubiquitinating enzyme (DUB) Usp14 is required for Wnt signaling (7) and these events can switch DVL between canonical and non-canonical pathways (8).

## DVL and development

Insights into the mechanisms of Wnt action have emerged from several fields of research: genetics in *Drosophila* and *Caenorhabditis elegans*; biochemistry in cell culture; and ectopic gene expression in *Xenopus* embryos. Wnt signaling is essential for mammalian embryogenesis (9,31) as well. Many Wnt genes in the mouse have been mutated, leading to specific developmental defects. The Wnt pathway acts as a regulator of cell patterning, proliferation, differentiation, cell-to-cell communication, adhesion and migration, cell survival, and apoptosis. It is required for normal development of some organs and organic systems, in particular the central nervous system through synaptic rearrangements (32). The Wnt pathway regulates the normal development of the neural plate, neural tube, brain, spinal cord, and sensory and motor neurons (9,33,34). In addition to neural tissues, Wnt pathway is critical for vascular and cardiac systems development and also modulates osteoblast physiology (35,36).

*Dsh* gene family has a significant role in the broad spectrum of developmental processes (3,37). *Dsh* alleles were first discovered in *Drosophila* mutants whose marginal wing bristles were “deranged and sparse” (38), and hence the name dishevelled. The allelic locus was rediscovered later on when it was shown that *dsh* was an important segment polarity gene in the early *Drosophila* embryo. *Drosophila* gene *dsh* was cloned in 1994 (39,40) and the first *Xenopus* homologue *dvl* in 1995 (41). The three murine homologues were cloned soon after (42-44), followed by cloning of three human *DVL* genes (3,17,18,43). The function of *dvl* homologues seems to have diverged among the vertebrates. The evolution of *dvl* homologues with their functional specificities is supported by the findings that in *Xenopus, dvl1* and *dvl2* homologues, but not *dvl3*, are necessary to mediate the Wnt-dependent signals that control neural crest specification. On the other hand, in rodents *Dvl2* and *Dvl3* are involved in neural crest development, but not *Dvl1* (5). The lack of *Dvl3* in mice affects the formation of the neural tube, heart, and inner ear (37). It is noteworthy that defects of these organs are much more severe when the mice are deficient in more than one *Dvl* family member. The role of *Dvl1* and *Dvl2* in somite segregation has also been investigated revealing that lack of these genes causes skeletal malformations in mice (45). Since the protein expression patterns during mouse development overlap, it seems that there are several developmental processes in which all three *Dvls* are functionally redundant. It is still not clear whether *Dvl*´s role in developmental processes is regulated through the canonical pathway, noncanonical pathway, or both (37).

In human developmental disorders, *DVLs* are reported only as candidate genes involved in certain syndromes, for instance the Schwartz-Jampel syndrome mapped to chromosome 1p36-p34 (17) and Charcot-Marie-Tooth disease type 2A mapped to 1p36-1p35 (17). It is also speculated that *DVL1* may have a role as a neural differentiation factor, which makes it a candidate gene for neuroblastomatous transformation. Bedell et al (46) suggested that *DVL* gene may play a role in the pathogenesis of the 1p36 deletion syndrome.

## DVL and cancer

The potential medical importance of Wnt signaling pathway has long been recognized in both human degenerative diseases and cancer. Many tumor types show high levels of beta-catenin and it is known that beta-catenin’s translocations to the nucleus indicate its acquisition of oncogenic activity. The mutations attributed to *APC, axin,* and *beta-catenin*, which encode components of the beta-catenin destruction complex are very common in a variety of investigated tumors (2,8). The constitutive activation of the Wnt pathway can lead to cancer (10) and *beta-catenin* can be proclaimed an oncogene. Since DVL protein is known as the central mediator of Wnt signaling, its inclusion in tumor formation has been under intensive investigation. DVLs are overexpressed in various tumor types, including lung cancer, prostate cancer, breast cancer, cervical squamous cell carcinoma, and gliomas (47-51). However, the importance of individual DVLs in tumor prognosis is in general poorly defined.

The functional consequences of the DVL family protein expression in tumor etiology are still not clear and the data reported are controversial. The majority of reports (49,50) indicate DVL overexpression and amplification, but there are also reports (50) on gross deletions of DVL loci. In primary lung cancer DVL3 is overexpressed in non small cell lung cancer, implying that these events upstream of beta-catenin are critical for activation of Wnt signaling (50). Surprisingly, overexpression of DVL1 or DVL2 was not detected. DVL3 mRNA is significantly higher in pleural effusions from patients with adenocarcinoma, suggesting that it might be used as a marker of pleural micrometastasis (49).

**Figure 3:**
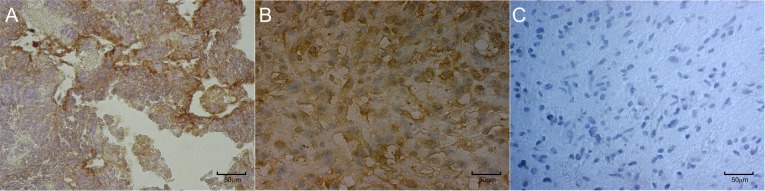
(**A**) Brain metastasis immunohistochemically stained for detection of dishevelled 3 (DVL3) protein (monoclonal mouse anti-human DVL3, Santa Cruz Biotehnology, Dallas, TX, USA), showing membranous staining. (**B**) Glioblastoma sample immunohistochemically stained for detection of dishevelled 1 (DVL1) protein (monoclonal mouse anti-human DVL1, Santa Cruz Biotehnology). (**C**) Control staining.

DVL family proteins are overexpressed in primary lung cancers (52) and the expression levels of DVLs were significantly higher in adenocarcinomas than in squamous cell carcinomas (48,53). The positive expression rates of DVLs are higher in stages III and IV tumors. Nodal metastases show higher expression levels of DVL1 and DVL3 than primary growths. However, the correlation with tumor prognosis has also not been established yet. Our investigations of DVL1 and DVL3 protein levels showed overexpression in brain metastasis of lung cancers (54) (Figure 3). Although reports indicate DVLs location in the nucleus to be only occasional (53), our study on brain metastases showed nuclear staining of both DVL1 and DVL3 proteins (54).

Breast cancers show aberrant expression of the *DVL1* gene (47). Amplification and up-regulation of *DVL1* gene are involved in breast carcinogenesis, especially in the acceleration of tumor growth. The involvement of DVL in invasive ductal carcinoma of the breast was also reported by Prasad et al (55). Mizutani et al (56) got similar results in prostate cancer. Their sample of 20 primary prostate cancer showed significant overexpression of DVL1 (65%). Correlation between DVL1 expression and beta-catenin expression was also confirmed. DVL2 is overexpressed in human high-grade gliomas, suggesting a role for active Wnt signaling in regulating the biology of these tumors (57). DVL1 is overexpressed in over two thirds of primary cervical squamous cell cancers when compared to corresponding non-cancerous uterine squamous cell tissues (58). Subsequently, amplification and increased expression of *DVL* genes may play an important role in the development of a portion of human cancers through derangement of the Wnt signaling pathway.

DVL is very much involved in invasion and metastasis of tumors – the so called epithelial-to-mesenchymal transition (EMT). The occurrence of EMT during tumor progression resembles the developmental scenario and sheds light on important mechanisms governing metastasis, where noninvasive tumor cells acquire motility and ultimately disseminate to places distant from the primary site. Wnt signaling pathway has a particularly tight link with EMT. Moreover, the stabilization and nuclear accumulation of beta-catenin can induce EMT (59,60) by activating the transcriptional repressors Snail and Slug that suppress E-cadherin expression thus inducing EMT (61). Moreover LEF 1 when overexpressed leads to enhanced tumor invasiveness and induces EMT (62-64). Subsequently DVLs too are involved in tumor metastasis (60,65).

## Conclusion and future perspectives

Ever since the first discovery of the *Dsh* allele in *Drosophila* mutants, dishevelled genes and proteins have been assigned the central role in the mediation of Wnt signaling. The Wnt signal utilizes DIX and PDZ or PDZ and DEP domains of DVL proteins to channel signal into canonical or non-canonical downstream pathways.

Recent data suggest that in the canonical pathway DVL is responsible for the disassembly of the beta-catenin destruction complex and recruitment of AXIN to the membrane, where DVL forms large molecular supercomplexes. DVL is considered to be a key regulator that rescues cytoplasmic beta-catenin from degradation.

DVL activity is dynamically regulated by phosphorylation, ubiquitination, and degradation and it seems to be dependable on the cellular context as well. Constitutive activation of the Wnt pathway can lead to cancer. The mutations attributed to *APC, axin,* and *beta-catenin*, which encode components of the beta-catenin destruction complex are very common in a variety of tumors.

Therefore, molecular components of Wnt pathway (like beta-catenin for instance) are relevant biomarkers helpful in better diagnosis and treatment. DVLs are overexpressed in various tumor types, including lung cancer, prostate cancer, breast cancer, cervical squamous cell carcinoma, and gliomas. However, the importance of individual DVLs in tumor prognosis still needs elucidation. The approaches to decrease DVL expression, as well as agents blocking selected specific DVL interactions, may be of particular interest as potential targets for therapeutic interventions.
